# Making sense of scattering: Seeing microstructure through shear waves

**DOI:** 10.1126/sciadv.adp3363

**Published:** 2024-07-31

**Authors:** Giacomo Annio, Sverre Holm, Gabrielle Mangin, Jake Penney, Raphael Bacquët, Rami Mustapha, Omar Darwish, Anna Sophie Wittgenstein, Katharina Schregel, Valérie Vilgrain, Valérie Paradis, Knut Sølna, David Alexander Nordsletten, Ralph Sinkus

**Affiliations:** ^1^ Laboratory of Vascular Translation Science, LVTS, U1148, National Institute for Health and Medical Research (INSERM), Paris, France.; ^2^ Department of Physics and Computational Radiology, Oslo University Hospital, Oslo, Norway.; ^3^ Department of Physics, University of Oslo, Oslo, Norway.; ^4^ Department of Radiology, Beaujon Hospital, Clichy, France.; ^5^ School of Biomedical Engineering and Imaging Sciences, King’s College London, London, UK.; ^6^ Department of Neuroradiology, Heidelberg University Hospital, Heidelberg, Germany.; ^7^ Inflammation Research Center, CRI, U1149, National Institute for Health and Medical Research (INSERM), Paris, France.; ^8^ Department of Pathology, Beaujon Hospital, Clichy, France.; ^9^ Department of Mathematics, University of California at Irvine, Irvine, CA, USA.; ^10^ Department of Biomedical Engineering and Cardiac Surgery, University of Michigan, Ann Arbor, MI, USA.

## Abstract

The physics of shear waves traveling through matter carries fundamental insights into its structure, for instance, quantifying stiffness for disease characterization. However, the origin of shear wave attenuation in tissue is currently not properly understood. Attenuation is caused by two phenomena: absorption due to energy dissipation and scattering on structures such as vessels fundamentally tied to the material’s microstructure. Here, we present a scattering theory in conjunction with magnetic resonance imaging, which enables the unraveling of a material’s innate constitutive and scattering characteristics. By overcoming a three-order-of-magnitude scale difference between wavelength and average intervessel distance, we provide noninvasively a macroscopic measure of vascular architecture. The validity of the theory is demonstrated through simulations, phantoms, in vivo mice, and human experiments and compared against histology as gold standard. Our approach expands the field of imaging by using the dispersion properties of shear waves as macroscopic observable proxies for deciphering the underlying ultrastructures.

## Teaser

In vivo imaging of multiply scattered shear waves on vessels reveals tissue’s microvascular architecture.

## INTRODUCTION

Waves have the potential to unveil insight into the structure and dynamics of matter, otherwise inaccessible. In particular, wave scattering processes have demonstrated the ability to characterize embedded ultrastructures in the context of light (*1*–*3*), acoustics (*4*, *5*), geophysics (*6*), and particle scattering (*7*). In complex disordered media, different models of wave propagation exist to describe the interaction between waves and scatterers (*8*–*10*). In the context of elastic scattering processes with short propagation path and low scatterer densities, the well-known Born approximation allows to describe the physics using only one single interaction between wave and scatterer. As the number of scattering events increases and waves propagate over several wavelengths within the medium, the single scattering approximation becomes invalid and multiple scattering processes must be taken into account (*11*).

Recently, the domain of shear wave imaging has received increased attention due to its ability to quantify noninvasively tissue mechanics for disease characterization in various organs (*12*, *13*). While this methodology has been very successful in quantifying the bare shear modulus (stiffness) (*14*–*17*), the origin of shear wave attenuation in tissue is currently not properly understood leading to a mismatch between theory and data when investigating the wave’s dispersive properties (*18*, *19*). Decrypting the physics of attenuation has the potential to extend and broaden the scope of shear wave imaging toward a method enabling to quantify at clinical imaging scales ultrastructures, i.e., vascular architecture, which is a key biomarker for many diseases in particular cancer (*20*–*23*). Here, we present a theory that overcomes this current limitation and demonstrate that it enables to harness information about micro vasculature architecture from macroscopic shear wave dispersion imaging.

## RESULTS

### Wave attenuation in tissue has a substantial scattering component

**Fig. 1.**
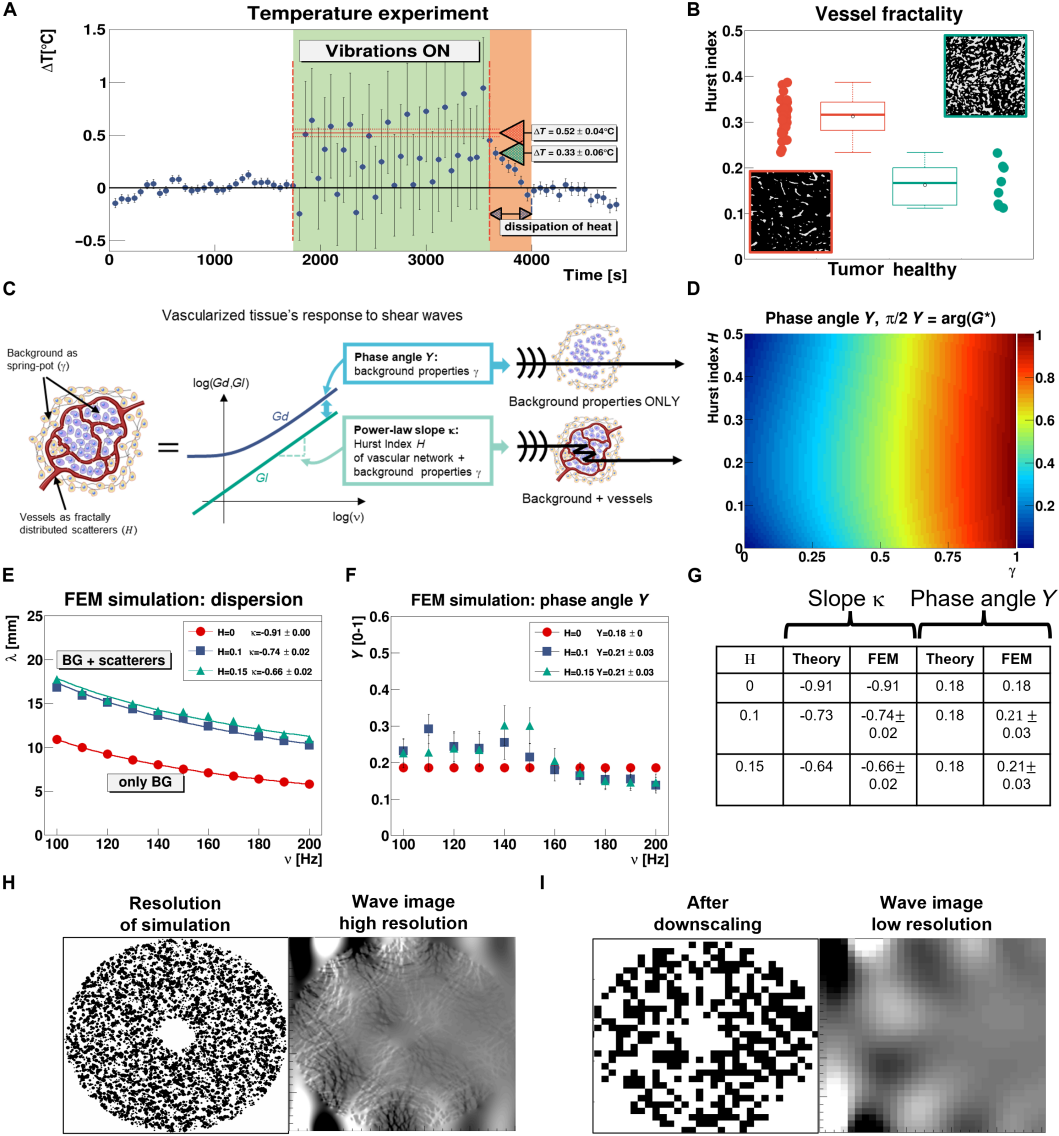
Shear viscosity has a substantial contribution originating from wave scattering onto vessels, and wavelength dispersion properties depend on the scatterers’ Hurst index. (**A**) Measured temperature shift in a bovine tissue specimen quantified via MRT due to 200-Hz mechanical vibrations. The green and red arrows indicate the measured and theoretical temperature increase, respectively, assuming for the latter that the material’s attenuation is purely due to absorption. (**B**) Box plot showing the Hurst index *H* of vessels in tumoral and healthy liver tissue (with corresponding segmented histological cuts). (**C**) Vascularized tissue is modeled as a composite material made from an effective background described by a single classical spring-pot model. The power law exponent (slope κ) is linked to vasculature’s Hurst index (through scattering) and tissue’s constitutive properties (γ), while the phase angle *Y* depends solely on the constitutive background properties (γ). (**D**) The phase angle *Y* is bound to the admissible interval [0 − 1] for all the possible combination of γ and *H*. (**E**) Wavelength dispersion of propagating shear waves using FEM simulations in 2D scattering structures exhibiting two different Hurst indices (*H* = 0.1,0.15) for a given background (γ = 0.18). Data points and solid lines indicate the estimated wavelengths and corresponding power law fits, respectively. (**F**) Corresponding dispersion of the phase angle *Y*. (**G**) Table showing theoretical and corresponding estimated values for κ and *Y*. (**H**) Simulation of multiple wave reflections at a very fine spatial resolution with corresponding wave fields (curl of the 2D wave field). (**I**) Downscaled version of the simulated data.

In general, shear wave attenuation is caused by two fundamentally different physical phenomena: absorption and scattering. The former is an energy dissipation mechanism, leading to a temperature increase within the material. In contrast, the latter is only an energy redistribution in space and time, linked to the scattering phenomenon without any concomitant heat exchange. Both processes, however, affect the wave’s dispersion behavior within the material as well as its modulus estimate from shear wave experiments. To investigate whether scattering is a prominent contribution to the dispersion in tissue, freshly excised bovine tissue was investigated using magnetic resonance thermometry (MRT) with and without shear vibrations (Fig. 1A, “Temperature experiment”). Here, ∆*T* represents the temperature difference between the specimen and a reference tissue sample that is not exposed to vibrations. During the first 1800 s of the experiment, the tissue reaches thermal equilibrium, and no statistically significant temperature shift is visible with respect to the reference (error bars correspond to 3σ). The sample is then exposed for 30 min to 200 Hz shear vibrations. Throughout vibrations (green background in Fig. 1A), MRT quantification is compromised due to the strong motion (~50-μm amplitude) (*24*). The first temperature measurement right after vibrations were turned off yields a temperature increase of ∆*T* = 0.33° ± 0.06°C. However, using the measured viscoelastic properties and attributing the entire viscous component to absorption processes, we should expect a ∆*T* = 0.52° ± 0.04°C. Mind that we also observe the dissipation of the energy (orange background in Fig. 1A) and the return to equilibrium once vibrations are turned off, as expected. The observed deviation indicates that roughly 40% of attenuation originates from scattering for this tissue sample, representing hence a substantial contribution. Consequently, a comprehensive rheological model for tissue needs to account for scattering, which is most likely the process at the root of the observed mismatch between theory and data seen in previous in vivo shear wave experiments (*18*, *19*).

### Tumor vasculature is “rough”

Vessels are the single one component in tissue at the mesoscopic scale for representing scatterers to shear waves. In general, vascular architecture can be characterized in many ways. One common metric is the Hurst index *H*, which quantifies the obstacles’ randomness (*25*), and can be calculated via the classical box counting approach (*26*). Architecturally, the Hurst index describes the rate at which the frequency distribution of spatial distances between pairs of vessels (lags) increases or decreases (*27*). *H* is directly related to the fractal dimension via *D_f_* = 2 − *H*, and shows distinct differences between the vascular organization of normal human liver tissue and hepatocellular carcinoma (Fig. 1B). The Hurst index was quantified from the shielded covariance function of vasculature segmented from immunohistological cuts stained for vessels (CD31, *P* < 10^−7^). Intriguingly, the observed range for *H* ∈ [0,0.5] indicates the regime of antipersistence, i.e., it characterizes a so-called “rough, wild” material (*25*), which is matching the regime for the theoretical description of wave scattering (*28*). A way to reveal such complex microstructures is to make use of wave-matter interaction via the process of anomalous wave propagation, which has already been used in the context of particle diffusion (*29*), light propagation (*3*), and effect on mechanical waves (*27*, *30*). However, the intricate link between the spatial organization of scatterers and the underlying constitutive material properties has been elusive so far for shear waves in tissue.

### Multiple reflections’ model disentangles scattering from constitutive mechanical properties

Microrheological shear wave experiments, performed on living cells devoid of blood vessels, yield findings consistent with a frequency power law, in accordance with the so-called rheological spring-pot model for over four decades of frequency (*31*). The spring-pot models the hierarchical organization of tissue via an infinite series of springs and dashpots with a distinct power law decay spectrum (*32*). While the fundamental drivers of this rheological behavior may exhibit more complexity, the spring-pot has shown remarkable promise as a model for viscoelastic response. The model proposes that the frequency dispersion of the complex-valued shear modulus G^*^ and the normalized phase angle Y=arg(G*) · 2π are controlled by a single parameter γ ∈ [0,1], which determines whether the material is behaving like a spring (γ ≈ 0) or rather like a dashpot (γ ≈ 1). It is expressed by $G^{*}=|G^{*}|e^{i\frac{\pi }{2}Y}\propto \omega^{\gamma }e^{i\frac{\pi }{2}\gamma }$ , and leads to an accepted model for the dispersion of the shear wave speed *C*_s_ in tissue (*33*), with ω the circular frequency. However, once vascularized tissue is considered, it fails to describe simultaneously the measured phase angle *Y* (*18*, *19*). Therefore, a comprehensive model for shear wave propagation in vascularized tissue needs to consider fractal-like scatterers representing the vessels embedded in a background material modeled according to the spring-pot (Fig. 1C). As discussed later, only vessels exhibiting smooth muscle cells on their wall act as scatterers.

Here, we propose to use the framework of the O’Doherty-Anstey (ODA) theory (*6*, *30*) to describe the scattering phenomena. So far, the classical ODA scheme described scattering in the presence of lossless background materials only. Moreover, within the ODA theory the spatial distribution of scatterers is characterized via its covariance function, which in our case, contrary to specific covariance functions used in the past (*27*, *28*), rises as a function of lag with the Hurst index. Such behavior is linked to the fact that shear waves probe only the geometry of the space between the scatterers, the space where shear waves travel and get multiply reflected. Shear waves do not propagate in liquid media and thus through vessels.

Our theoretical model, based on first principles, predicts that the dispersion of the shear wavelength ∣λ∣ evolves according to $|\lambda |\propto \omega^{\kappa }=\omega^{-1+\frac{\gamma }{2}+2H-\gamma H}$ , and that the phase angle only depends on the constitutive background material, i.e., *Y* = γ (“A model for wave scattering” section and Fig. 1C). Hence, experimental quantification of κ and *Y* at the spatial scale of the wavelength allows to infer the underlying microarchitectural effective Hurst index *H*, governing the scatterer distribution. We use, as mentioned in Materials and Methods, effective Hurst index and Hurst index synonymously. Thereby, microstructure becomes visible macroscopically. The phase angle *Y* is properly bound to the interval [0 − 1], whereby avoiding an unphysical material that has negative elasticity (*Y* > 1) or generates energy (*Y* < 0) (Fig. 1D). Furthermore, the slope of the wavelength’s dispersion, κ, is bound to the range [−1,0] for the permissible ranges of γ ∈ [0,1] and *H* ∈ [0,0.5]. Values for *H* > 0.5 would imply a positive value for κ, hence describing a rather uncommon material where the wavelength increases with frequency. Keep in mind that our results obtained from histopathology (Fig. 1B) indicate an antipersistent regime for *H*, allowing the dispersion properties of $G^{*}\left(\omega \right)∼{C_{s}}^{2}$ to explore the entire permissible range of its power law exponent from [0,2] (*34*).

It is important to notice that the validity of the ODA framework holds only for the regime where the wavelength is large compared to the mean free path between scatterers. At higher frequencies (thus shorter wavelengths) we enter the “ballistic” regime where the wave starts to resolve the spaces between the scatterers and the dispersion begins to depend solely on the constitutive properties of the background. Contrary to the classical ODA theory, in our model, the onset of the multiple reflections’ regime is not dependent on the number of wavelengths the wave has traveled through the material [chapter 9.3.2. p. 267 in (*35*)].

### FEM simulations confirm model predictions

Finite element model (FEM) simulations (*36*) of shear wave experiments performed for different fractal distributions of scatterers (Fig. 1, E and F) confirm the theoretical predictions for slope κ and phase angle *Y* of the proposed model (Fig. 1G). Here, the background material was modeled according to the spring-pot model with a fixed value for γ, and the scatterers as objects 10 times stiffer to represent strong reflectors for shear waves. The multiple reflections’ behavior of scattered shear waves was replicated by simulating their propagation at a very fine spatial resolution with a subsequent down sampling of the spatial wavefield. The down sampling step allows to model the conditions of a real imaging experiment, where individual voxels are large (in the millimeter range) compared to the typical distance between scatterers (in the micrometer range) (Fig. 1, H and I). At coarse spatial resolution, the fine details of the scattering structure are not resolvable anymore and the material behaves like an effective medium. In general, recovery of local biomechanics from the wave field, i.e., shear wavelength λ, shear wave speed *C*_s_, and phase angle *Y*, is done via established inversion methods (*30*, *37*, *38*). Dispersion results match the prediction of the theoretical model, i.e., the wavelength follows a power law exponent according to ∣λ∣~ω^κ^, $\kappa =-1+\frac{\gamma }{2}+2H-\gamma H$ , and a frequency-independent phase angle depending only on the background properties, i.e., *Y* = γ.

### Scattering quantifies the Hurst index in 3D-printed fractal structures

**Fig. 2.**
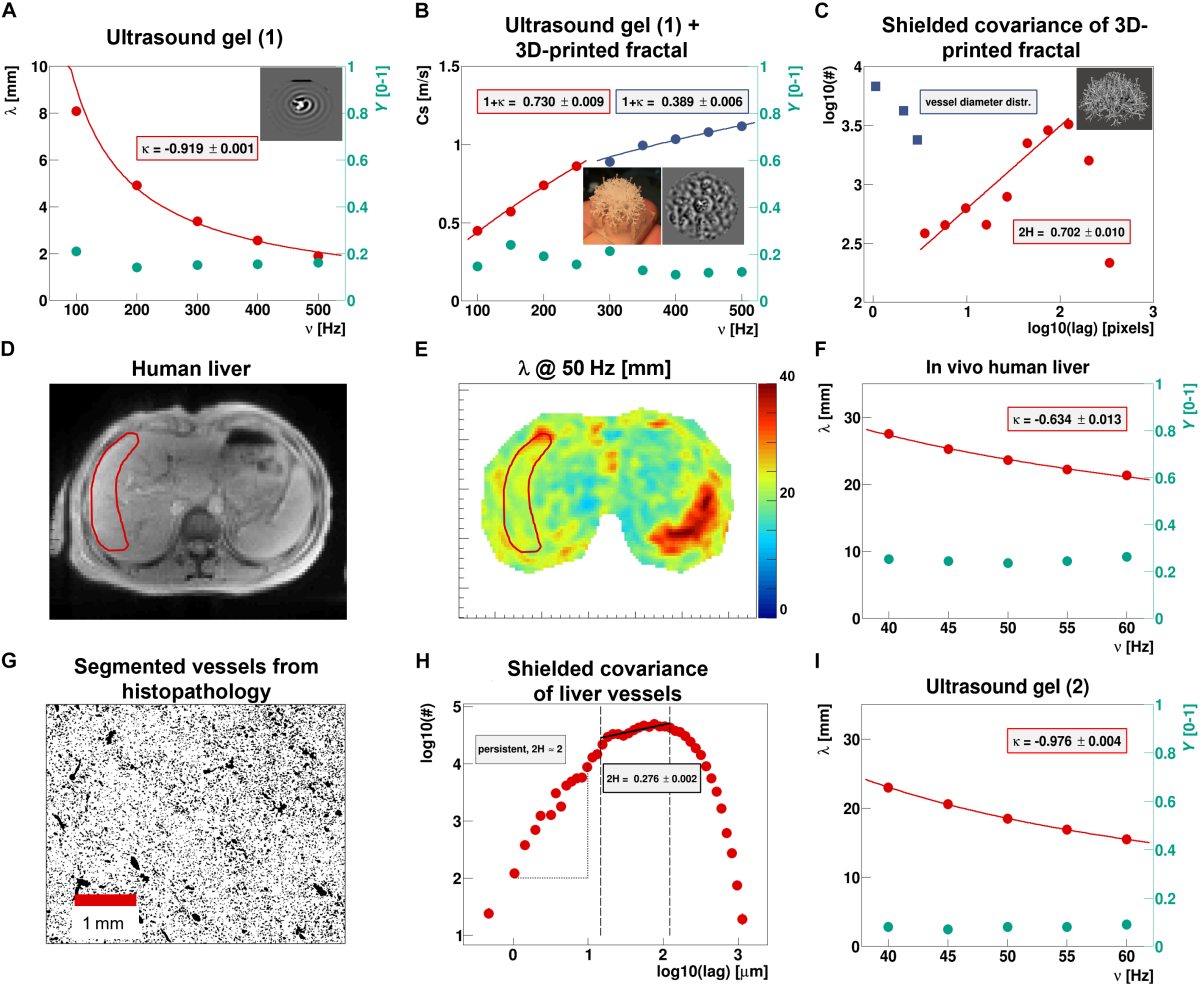
3D scattering structures, described by their Hurst index, affect differently the dispersion of wavelength and phase angle, shown in vivo and in vitro. (**A**) The dispersion of shear wavelength (red dots, left *y* axis) and phase angle (green dots, right *y* axis) in an ultrasound gel (1) are presented; the inset shows the corresponding experimental shear waves. (**B**) The presence of a 3D-printed fractal structure (3D print and corresponding shear wave pattern shown in the insets) within the ultrasound gel affects the shear speed’s dispersion (red and blue dots) but not the phase angle’s (green dots). A power law fit (with corresponding fit parameters) is shown for each of the two frequency regimes (multiple scattering in red, ballistic in blue). (**C**) Shielded covariance of the 3D-printed fractal structure (in the inset) showing a sharply falling distribution centered at very small diameters (in blue) and a rising part probing the lags between the scatterers (datapoints and fit are indicated in red). (**D**) MRI image of a healthy volunteer’s liver. (**E**) Corresponding shear wavelength map at 50-Hz vibrations. (**F**) Wavelength’s (red dots, left *y* axis) and phase angle’s dispersion properties (green dots, right *y* axis) within the liver region of interest (ROI) (red ROI in D) with corresponding frequency power law fit. (**G**) Section of healthy human liver tissue stained (CD31) and segmented for vessels. (**H**) Corresponding shielded covariance. (**I**) Dispersion properties of the wavelength (red dots, left *y* axis) and phase angle (green dots, right *y* axis) in ultrasound gel (2) follow the spring-pot model.

Multifrequency shear wave imaging was performed via magnetic resonance elastography (MRE) in the frequency range from 100 to 500 Hz in a sample of ultrasound gel using a preclinical high-field (7 T Bruker) magnetic resonance imaging (MRI) system (Fig. 2A) (*37*). In short, MRE uses a phase-sensitive MRI sequence phase-locked to an externally elicited mechanical vibration to enable the noninvasive visualization of propagating shear waves (*12*). Knowledge of the three-dimensional (3D) complex-valued displacement field allows the local calculation of the complex shear modulus by inverting the wave equation, with shear wavelength typically much larger than the voxel size (*18*, *19*).

Ultrasound gel is used as its stiffness is in the range of living tissue (~1 kPa) and *H* = 0. Here, the measured dispersion of the wavelength yields a power law exponent κ = −0.92 ± 0.001, which should match $-1+\frac{\gamma }{2}$ assuming the validity of the spring-pot model. We observe a perfect agreement with the corresponding phase angle via $\frac{Y}{2}=0.081\pm 0.001$ , which assumes γ = *Y*. Once a 3D-printed fractal-like scattering structure is added to the gel, the phase angle is unaffected ( $\frac{Y}{2}=0.081\pm 0.001$ ), while the wave speed’s dispersion changes substantially (Fig. 2B). The shear speed dispersion shows two different regimes: one low-frequency regime dominated by multiple scattering and one high-frequency regime where the ballistic solution is approached. At low frequencies, (100 to 250 Hz), the wave speed’s dispersion 1 + κ = 0.73 ± 0.01 and the phase angle via $\frac{Y}{2}=0.09\pm 0.01$ are no longer consistent with the spring-pot model. However, these data are in line with our theoretical framework, meaning we are in the multiple reflections’ regime. The combined information of speed dispersion and phase angle leads to an estimated Hurst index $\left(H=\frac{1+\kappa -\frac{\gamma }{2}}{2-\gamma }\right)$ of 2*H* = 0.7 ± 0.02, which agrees very well with its estimation from the a priori known 3D-printed structure’s shielded covariance function providing 2*H* = 0.702 ± 0.01 (Fig. 2C). We use 2*H* instead of *H* as it relates directly to the rising part of the covariance function (“A model for wave scattering” section and Eq. 10). At higher frequencies (300 to 500 Hz) the speed dispersion starts to flatten out, approaching the spring-pot predictions, i.e., $1+\kappa \to \frac{\gamma }{2}$ . In this case, the wavelength starts to approach the lag distance and the wave thus probes solely the underlying material properties, i.e., the ultrasound gel.

### In vivo estimated Hurst indices match histology

Shear wave dispersion was quantified in the liver of healthy volunteers (*n* = 10) at the organ level via clinical MRE (3 T, SIEMENS Healthineers) in the frequency range from 40 to 60 Hz (*38*) (Fig. 2, D to F). The measured slope for the shear wavelength of κ = −0.63 ± 0.01 is not matching the corresponding phase angle $\frac{Y}{2}=0.125\pm 0.01$ via $-1+\frac{\gamma }{2}$ as predicted by the classical spring-pot model. The combined information of κ and γ yields an estimated Hurst index of 2*H* = 0.275 ± 0.02 for the vasculature of healthy liver parenchyma.

In humans, blood vessels with a diameter above approximately 10 − 20 μm exhibit smooth muscle cells (*39*) rendering the vessel wall much more rigid (in the megapascal range) than the other components of tissue (in the kilopascal range). They can henceforth be considered as scatterers. Figure 2G shows a histological image of healthy liver tissue, stained (CD31), and segmented for blood vessels. Here, structures below 15 μm were removed since they represent capillaries and therefore do not act as shear wave scatterers. The corresponding shielded covariance (Fig. 2H), shows three distinct regimes: one regime below 15 μm (left vertical dashed line) describing surface roughness of the structures, in this case characteristic of a persistent fractality (*H* > 0.5), one above 15 μm, rising with a Hurst index 2*H* = 0.27 ± 0.002, indicative for an antipersistent regime (*H* < 0.5), thus characterizing the space between vessels, and one dropping with increasing lag (right vertical dashed line). The fit has been done to the entire intermediate regime to obtain the least biased result, yielding a Hurst index, which is in very good agreement with the in vivo estimate from shear wave scattering. Mind that the slope of the covariance function below 15 μm exhibits a value of 2*H* ≈ 2, which resides in the persistent regime of the Hurst index (*H* ∈ [0.5,1]), very different to blood vessels. This is indicative for structures that are not “rough” but instead follow a trend. As a cross-validation step, MRE experiments were performed in a second ultrasound gel phantom under identical clinical experimental conditions. Dispersion results for this gel-like type of material confirm again the validity of the spring-pot model, as expected (Fig. 2I), in contrast to the in vivo results obtained in human liver (Fig. 2F).

**Fig. 3.**
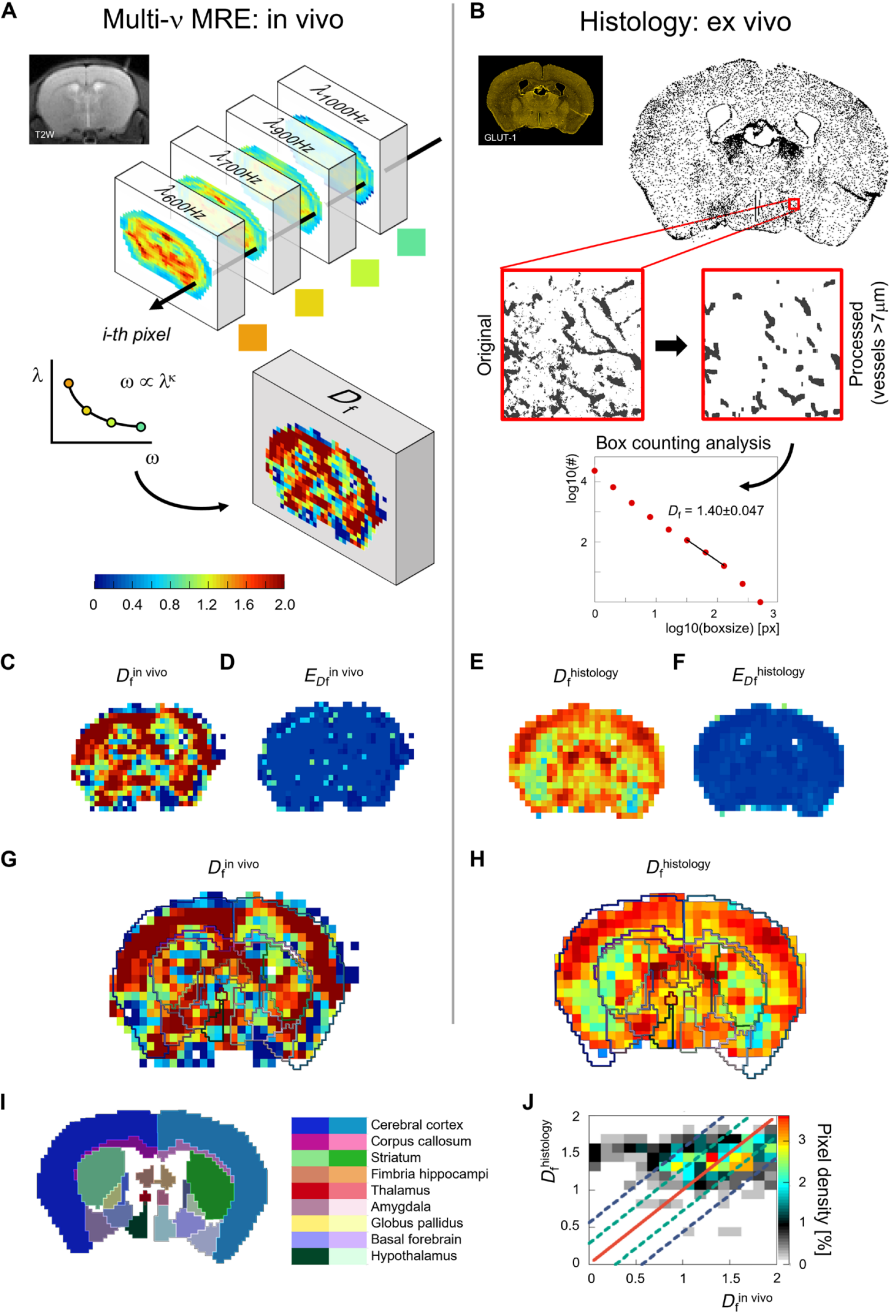
Maps of vascular fractality in the mouse brain, obtained in vivo via shear wave scattering and ex vivo from histology, show a strong correlation and match the anatomical atlas. (**A**) Individual MRE experiments in a living mouse provide spatially resolved images of the shear wavelength for each mechanical excitation frequency. A pixel-wise power law fit to the dispersion properties and an average of the phase angle obtained from all frequencies allows to extract for each image pixel the corresponding Hurst index *H*, as explained beforehand. This yields spatially resolved maps of the vasculature’s fractality via *D*_f_ = 2 − *H*. (**B**) Ex vivo box counting analysis pipeline: vessels were stained (Glut1) on tissue slices, and corresponding images accordingly segmented. Structures below 7 μm in size were removed as they constitute capillaries in mice and therefore do not exhibit smooth muscle cells. Box counting was performed on sub-tiles of 512 × 512 pixels. (**C**) Map of *D*_f_ from in vivo shear wave scattering ( $D_{f}^{\text{in vivo}}$ ). (**D**) Corresponding error ( $E_{Df}^{\text{in vivo}}$ ). (**E**) Map of *D*_f_ from histology ( $D_{f}^{\text{histology}}$ ). (**F**) Corresponding error ( $E_{Df}^{\text{histology}}$ ). (**G**) $D_{f}^{\text{in vivo}}$ coregistered to a mouse brain atlas with anatomical regions overlaid. (**H**) Corresponding map of $D_{f}^{\text{histology}}$ . (**I**) Matching section of a mouse brain atlas. (**J**) Correlation between in vivo and histology derived fractal dimension as a pixel density plot with respect to the total number of pixels in the brain region. The solid red line represents unity, while the dashed green (blue) lines indicate SD(s) of ±1σ (±2σ), respectively.

To further challenge our theory, we used our model to provide a characterization of mouse brain vasculature organization at the pixel scale. A healthy mouse underwent multifrequency MRE from 600 to 1000 Hz at 250-μm isotropic imaging resolution to assess shear wave dispersion within the brain (Fig. 3A) (*17*). The Hurst index obtained from imaging was converted to fractal dimension via *D*_f_ = 2 − *H* and compared to results obtained from local box counting on corresponding histological images stained for blood vessels (GLUT1), subsequently segmented (Fig. 3B). Structures below 7 μm size had been removed as they constitute capillaries in mice and therefore do not exhibit smooth muscle cells (*40*). *D*_f_ was quantified from a fit to the box size range of [32 to 128 pixels] probing the range from ~20 to 80 μm, which corresponds to the range where cortical vascular networks have scale-invariant fractal properties (*41*). The maps of vessel fractality *D*_f_ and their corresponding errors *E*_*D*f_ are presented in Fig. 3 (C to F), for in vivo and histology, respectively. Considering that we are bridging three orders of magnitude, millimeter shear wavelength probing micrometer structures, the maps show impressive similarity in the anatomical distribution of the areas with low/high vascular fractality.

Furthermore, maps of vessel fractality were coregistered to a mouse brain atlas (*42*) applying a rigid transform using the open-source 3D Slicer software (Fig. 3, G to I) (*43*). Here, it can be appreciated that *D*_f_ varies with respect to brain regions similarly between both methods, e.g., the cortex as a predominant gray matter structure (blue labels) has a higher fractality than the corpus callosum (pink labels), which is the largest murine white matter tract. Deep grey matter structures such as the striatum (green labels), the thalamus (red labels), or the globus pallidus (yellow labels) that are interspersed with white matter tracts such as the internal capsule or the corticospinal tract (not segmented, as they are beyond the spatial resolution of our sequences), show a more heterogeneous pattern of *D*_f_. The elevated fractal dimension close to 2 in the cortex, visible both in vivo and in histology, might stem from the hierarchical organization of leptomeningeal blood vessels that radially enter the cortex from the surface. White matter tract structures as the corpus callosum have longer but sparser vascular and microvascular branches compared to gray matter area (cortex and basal ganglia), which likely justifies the regional differences in the fractality (*44*). Figure 3J shows the correlation between in vivo and histology derived fractal dimensions (both coregistered to the atlas space) as a pixel density plot with respect to the total number of pixels. The comparison is done by using the average fractal dimension of a sliding window of 3 × 3 pixels for both quantities to account for a coregistration mismatch of about ±1 pixel between the two images. We observe a good correlation with approximately 62% (82%) of pixels matching within ±1σ (±2σ) SD(s), respectively. Of course, one expects that there will be some differences in coalignment and warping comparing live-animal imaging and ex vivo histology. Moreover, $D_{f}^{\text{histology}}$ is measured on slices 30 μm in thickness contrasting the MRE acquisition which integrates over 250 μm, making precise quantitative comparison between methods challenging.

## DISCUSSION

Elastic wave scattering carries the intrinsic opportunity to unravel microscopic ultrastructures from macroscopic observations. Despite recent advances in shear wave imaging, a thriving new imaging branch for the characterization of tissue biomechanics, such possibility was hindered by a missing fundamental understanding of shear wave propagation in the multiple scattering domain.

In a first step toward achieving that objective, we understood via an MRT experiment, that tissue’s attenuative behavior originates to a substantial degree from scattering, shown by the mismatch between theoretically expected and measured thermal heating during shear wave experiments. This fundamental finding demonstrates the necessity to consider scattering in the theoretical description of shear wave propagation in tissue. Vessels exhibiting smooth muscle cells are the prime and only candidates for scatterers within tissue due to their stiff outer shell and fractal-like architecture.

The next step was to provide the mathematical and theoretical framework to understand the physical origin of this phenomenon in the context of the ODA theory, which has been originally developed in geophysics. This theory also leverages the use of the spring-pot (or fractional viscoelasticity) as a model of the frequency response of materials. While this model may simplify the broader material response across many decades of frequency, it has shown promise as a model in the examined frequency range. Our framework allows to extract from macroscopic shear wave dispersion imaging the underlying microscopic parameters, which characterize on the one hand the background material, and on the other hand, the architecture of the vasculature.

We demonstrated via experiments, that once scattering structures are added to a classically behaving gel, the currently established rheological spring-pot model breaks down. Our model not only correctly describes the biomechanics of such complex media but it also allows to estimate the architectural index of the scatterers. The investigation of spatially varying fractality in 3D-printed structures was not possible due to the limited resolution of our 3D printer. Furthermore, the validity of our model is demonstrated in vivo by showing that the architectural index of healthy liver vasculature matches corresponding histology. This finding holds on the organ level and yields, due to the large statistics, excellent agreement with the ground truth.

In a final step, we went beyond assessing spatially homogeneous vasculature organization in 3D-printed fractals or homogeneous liver tissue. We compared spatially resolved maps of microvasculature in healthy mouse brain, quantified pixel-wise at 250-μm pixel level via shear wave dispersion imaging, to the corresponding architectural index derived from histology. The overall agreement, both spatially and quantitatively, is notable. When coregistered to a mouse brain atlas, gray and white matter regions show their differences in vascular architecture as expected from histology.

In comparison to other existing techniques, multifrequency MRE does not require any contrast agent; instead, it relies solely on the interactions between waves and vessels, whether perfused or not. This makes the wave scattering approach robust against perfusion heterogeneities, often characterizing tissue in the presence of diseases. Accessing noninvasively a 3D map of microvasculature architecture opens the gateway to the translation of this method to clinical application. Considering the undoubted importance of vasculature in many diseases such as cancer, this method could potentially allow to disentangle treatment effects on different components of the tumor microenvironment in the context of therapies.

## MATERIALS AND METHODS

### Temperature experiment

We estimate the expected temperature rise ∆*T* from the heat-transfer equation, where we assume for simplicity a planar shear wave and the viscosity tensor η_iklm_ linked to the imaginary part of the complex shear modulus for the isotropic case via η = G_l_/ω

$$\rho C_{P}\frac{\Delta T}{\Delta t}=Q_{h}=\frac{1}{2}\omega^{2}\eta_{\text{iklm}}\epsilon_{\text{ik}}\epsilon_{\text{lm}}=\frac{1}{2}\omega G_{l}\epsilon^{2}$$ (1)

where *Q*_h_ represents the internal work within a viscoelastic material and we ignored any spatial dissipation [chapter 34 (*45*)]. In the above expression, ρ = 1000 kg/m^3^ is the density of tissue, *C*_P_ ≈ 3.6 kJ/(kgC) the specific heat capacity of tissue, ω = 2π · 200 Hz the angular frequency at which the experiment was performed, *G*_l_ = 3.26 kPa the measured loss modulus of the tissue sample, ε = *A* *k* the shear strain calculated from the measured amplitude *A* = 48 μm and the *k* vector *k* = ω/*C*_s_ of the sinusoidal vibration, and *C*_s_ = 2.26 m*/*s the speed of the shear wave within this sample. After an exposure time of ∆*t* = 30 min, we hence expect to see a temperature increase of

$$∆T=∆t\cdot \frac{\frac{1}{2}\omega G_{l}\varepsilon^{2}}{\rho C_{P}}\approx 0.52\pm 0.04{}^{\circ}C$$ (2)

assuming that the measured loss modulus within the sample is characterizing solely dissipative losses. The error corresponds here to 3σ.

### A model for wave scattering

We start with an established theory for a lossless background medium, where it is only the fractal pattern of scatterers that influences the wave’s amplitude and phase. This medium model is a refinement of the amplitude-model of O’Doherty and Anstey (*6*) as found by Garnier and Solna (*27*). This model will be amended in two important ways:

1) The background medium will be described by a fractional spring-pot rather than be lossless. The viscoelasticity is characterized by the order of the spring-pot, γ ∈ [0,1], where γ = 0 describes a lossless elastic medium and γ = 1 denotes a viscous, Newtonian medium.

2) The property of the fractality, described by the Hurst exponent, *H* ∈ [0,0.5], will be changed. The original theory builds on long-range correlation where the spatial correlation falls monotonically with a power law for large lags. Here, we allow for a rising correlation at short lags and then eventually a fall-off for large lags.

It is assumed that the background medium can be described by a fractional Newton model [chapter 5.6 (*35*)] where the strain-stress relations in time and frequency are

$$\sigma \left(t\right)=\eta \tau^{\gamma }\frac{\partial^{\gamma }\varepsilon \left(t\right)}{\partial t^{\gamma }}\Leftrightarrow G\left(\omega \right)=\frac{\sigma \left(\omega \right)}{\varepsilon \left(\omega \right)}=\eta {\left(i\omega \tau \right)}^{\gamma }$$ (3)

where ητ is a viscosity in Pa · s, τ = 1 is a reference time in seconds for nondimensionalization (mind that our final results do not depend on τ), and G(ω) is the complex shear modulus. Let a frequency-dependent phase velocity be *c*^2^ = *G*(ω)/ρ_0_, where ρ_0_ is the average density, and a characteristic phase velocity be c02=ηρ0 . The dispersion relation for a propagating wave in the background medium is then

$$k_{B}\left(\omega \right)=\frac{\omega }{c}=\sqrt{\rho_{0}}\omega G^{-\frac{1}{2}}\left(\omega \right)=l\omega^{1-\frac{\gamma }{2}}e^{-i\frac{\pi }{4}\gamma },\;l=\frac{\tau^{-\frac{\gamma }{2}}}{c_{0}}$$ (4)

Keep in mind that Eq. 4 uses the identity $i=e^{i\frac{\pi }{2}}$ . In such a medium, a wave will only travel a few wavelengths as given by the penetration depth

$$\delta_{p}=\frac{1}{2\pi }\frac{1}{\text{tan}\left(\frac{\pi \gamma }{4}\right)}$$ (5)

As an example, γ = 0.1 means that the wave will travel about δ_p_ = 2 wavelengths before its amplitude has fallen by *e*^−1^, and approximately 5 to 10 wavelengths before the energy is so low that it cannot be used for probing the medium anymore.

The propagation factor *e*^−*ikz*^ is a function of wave number, *k*, and distance, *z*. In a lossless background described by *k*_B_(ω) = ω/*c*_0_, due to the scattering [chapter 9.3.1 (*27*, *35*)] in the fractal, the wave number is

$$k\left(\omega \right)=i\frac{k_{B}^{2}\left(\omega \right)\;\phi \left(\omega \right)}{8}$$ (6)

The spatial correlation function of the random process due to the fractal scatterers is ϕ(*z*), with *z* spatial coordinate, and its frequency spectrum is

$$\phi \left(\omega \right)=2\int_{0}^{\infty }\;\phi \left(z\right)e^{-2ik_{B}\left(\omega \right)z}\;\mathrm{dz}$$ (7)

The model should satisfy the criterion that the real and imaginary parts of G^*^(ω), i.e., the elasticity and the viscosity, describe a realistic medium and therefore both be nonnegative for all values of ω.

Although derived originally for the lossless case, it can be shown that when the damping is strong and the wave propagates only a few wavelengths as indicated by Eq. 5, and there is scale separation between the spatial variation of the medium’s fluctuations and the wavelength, the wave number of the lossy background material of Eq. 4 can be substituted for *k*_B_(ω) in Eq. 6 giving the wave number as

$$k\left(\omega \right)=i\frac{l^{2}}{8}\phi \left(\omega \right)\omega^{2-\gamma }e^{-i\frac{\pi }{2}\gamma }$$ (8)

Likewise, the background wave number can be substituted in Eq. 7.

**Fig. 4.**
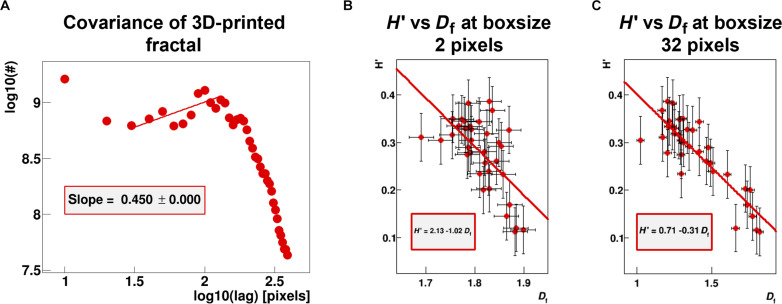
The Hurst index estimated from the shielded covariance, correlates to the fractal dimension *D*_f_. (**A**) Covariance function of medium fluctuations generated from the 3D-printed fractal-like structure shown in Fig. 2 (B and C). Mind that the full covariance includes lags which are not accessible to the process of multiple reflections as they are shielded. (**B**) Correlation of effective Hurst index *H*′ with corresponding fractal dimension *D*_f_ at box size 2 pixels, calculated for the data collective from Fig. 1A. (**C**) Corresponding correlation of *H*′ with *D*_f_ quantified at box size 32 pixels.

Medium fluctuations are introduced in ϕ(ω) via the covariance function of the scatterer distribution, ϕ(*z*). Figure 4A depicts the covariance function (not the shielded) for the analytic obstacles generated for the 3D-printed fractal-like structure shown in Fig. 2 (B and C). At small distances, the covariance basically probes the diameter distribution of the scatterers. The subsequent rise expresses the lags between the scatterers with the expected exponential drop at larger scales.

The covariance function can hence be parameterized by the sum of two functions: an exponentially falling part at small scales characterizing the diameter distribution of the scattering structure (blue squares in Fig. 2C), and a rising part according to the Hurst index *H* which gets eventually suppressed exponentially

$$\phi \left(z\right)=e^{-\alpha z}+\mu z^{2H}e^{-\beta z}$$ (9)

The initial falling part can be replaced by a spatial impulse function δ(*z*) as in our case, the shear wave dispersion within the scatterers is not quantifiable with our imaging method (either because of lack of any MRI signal in case of the 3D-printed fractal or due to moving liquid that does not support shear waves within real vessels) and thus does not contribute to our findings at all. Furthermore, the covariance function is mathematically correlating lags which are physically not accessible in our case due to the presence of strong reflection coefficients. We therefore will consider for the covariance function only the initial lags once an obstacle gets hit (shielded covariance) when we compare scatterer distributions to shear wave dispersion data. This “effective Hurst index” *H*′ correlates to the fractal dimension *D*_f_ quantified via classical box counting according to the expected law for the theoretical Hurst index, i.e., *H*′ ≈ 2 − *D*_f_ (Fig. 4B) using the histological data presented in Fig. 1A. Here, *D*_f_ was evaluated at the smallest scales, i.e., box size of two pixels. At larger box sizes, since the fractality of the vascular system changes over scale, that linear relationship is modified (Fig. 4C). For the sake of simplicity, we will use *H* throughout the paper as equivalent for the effective Hurst index *H*′. We therefore consider the following covariance function

$$\phi \left(z\right)=\delta \left(z\right)+\mu z^{2H}e^{-\beta z}$$ (10)

where μ ensures that ϕ(*z*) is positive definite such that the corresponding spectrum is nonnegative, *z*^2*H*^ models the rise of the shielded correlation function over a certain range of lags, and *e*^−β*z*^ tempers the rise so that the correlation asymptotically approaches 0 for large lags (Fig. 2, C and H). We remark that compared to Eq. 9, the expression in Eq. 10 is an idealization in the case with a rapid decay of correlation at the origin and a smoothing corresponding to a power spectrum vanishing for high frequencies. The integral of Eq. 7 is now

$$\phi \left(\omega \right)=1+2\mu \int_{0}^{\infty }z^{2H}e^{-\beta z}e^{-2i\omega^{1-\frac{\gamma }{2}}e^{-\frac{i\pi \gamma }{4}z}}\mathrm{dz}=1+2\mu \int_{0}^{\infty }z^{2H}e^{-\mathrm{az}}e^{\mathrm{ibz}}\mathrm{dz}$$ (11)

where the real and imaginary exponents contain these factors respectively

$$a=\beta +2l\;\text{sin}\left(\frac{\pi }{4}\gamma \right)\omega^{1-\frac{\gamma }{2}},\;b=2l\;\text{cos}\left(\frac{\pi }{4}\gamma \right)\omega^{1-\frac{\gamma }{2}}$$ (12)

The Fourier transform of the correlation function is as follows [chapter 3.944, equations 5 and 6 (*46*)]

$$\phi \left(\omega \right)=1+\mu \frac{\Gamma \left(2H+1\right)}{{\left(a^{2}+b^{2}\right)}^{H+\frac{1}{2}}}\;e^{\left[-i\left(2H+1\right){\text{tan}}^{-1}\frac{b}{a}\right]}$$ (13)

The first term relates to propagation processes within the scatterers, and, as discussed beforehand, does not affect our measured signals. Thus, it can be ignored here. The realistic medium condition mentioned in the previous section now has to be satisfied also for this more complicated model. That will enable us to determine the tempering factor β.

For both the real and the imaginary parts of G^*^(ω) to be nonnegative, the phase angle of G^*^ must be in the first quadrant. The corresponding properties can be found from those of the wave number as

$$G^{*}\left(\omega \right)=\frac{\rho_{0}\omega^{2}}{k^{2}\left(\omega \right)}$$ (14)

Our data are quite narrowband, and at the center frequency, ω_0_, only a β that has the same frequency dependency as *a* and *b* and with a factor of proportionality of 4*lH* ensures a phase angle in the first quadrant, i.e.

$$\beta =4\mathrm{lH}\omega_{0}^{1-\frac{\gamma }{2}}$$ (15)

as Fig. 1D illustrates.

For the special case of *H* = 0, this value of β will lead to

$$\phi \left(\omega_{0}\right)=\mu \frac{\Gamma \left(1\right)}{2l\omega_{0}^{1-\frac{\gamma }{2}}}e^{-i\;{\text{tan}}^{-1}\left[\text{cot}\left(\frac{\pi }{4}\gamma \right)\right]}=\frac{\mu }{2l\omega_{0}^{1-\frac{\gamma }{2}}}e^{-i\left(\frac{\pi }{2}-\frac{\pi }{4}\gamma \right)}$$ (16)

and from Eqs. 4 and 6

$$k\left(\omega_{0}\right)=i\frac{l^{2}\omega_{0}^{2-\gamma }}{8}e^{-i\frac{\pi }{2}\gamma }\cdot \frac{\mu }{2l\omega_{0}^{1-\frac{\gamma }{2}}}e^{-i\left(\frac{\pi }{2}-\frac{\pi }{4}\gamma \right)}=\frac{\mu l}{16}\omega_{0}^{1-\frac{\gamma }{2}}e^{-i\frac{\pi }{4}\gamma }$$ (17)

i.e., the classical spring-pot dispersion relation, as expected. This result will be used for finding the series expansion for the phase of G^*^ below. The results above inserted in Eq. 8 using the second part of Eq. 13 give for the magnitude of the wave number

$$|k\left(\omega_{0}\right)|\propto \omega_{0}^{1-\frac{\gamma }{2}-2H+\gamma H}$$ (18)

The wavelength will therefore depend on frequency as

$$\lambda =\frac{2\pi }{|k\left(\omega \right)|}\propto \omega_{0}^{\kappa },\;\kappa =-1+\frac{\gamma }{2}+2H-\gamma H$$ (19)

The power law exponent, κ, describes the wavelength’s dependency on frequency. The result for the phase of the wave number is

$$\text{arg}\left[k\left(\omega_{0}\right)\right]=\frac{\pi }{2}-\frac{\pi }{2}\gamma -\left(2H+1\right){\text{tan}}^{-1}\left[\frac{\text{cos}\left(\frac{\pi }{4}\gamma \right)}{2H+\text{sin}\left(\frac{\pi }{4}\gamma \right)}\right]$$ (20)

The properties of the complex shear modulus can be found from those of the wave number by Eq. 14. Its phase is accordingly

$$\begin{array}{cc} \text{arg}\left[G^{*}\left(\omega_{0}\right)\right] & =-2\text{arg}\left(k\right)\\ & =-\pi +\pi \gamma +2\left(2H+1\right){\text{tan}}^{-1}\left[\frac{\text{cos}\left(\frac{\pi }{4}\gamma \right)}{2H+\text{sin}\left(\frac{\pi }{4}\gamma \right)}\right] \end{array}$$ (21)

This expression can be expanded in a Maclaurin series about *H* = 0 giving

$$\text{arg}\left[G^{*}\left(\omega_{0}\right)\right]\approx \frac{\pi }{2}\gamma +H\left[-\pi +\frac{\pi }{2}\gamma +2\text{cos}\left(\frac{\pi }{4}\gamma \right)\right]$$ (22)

When comparing the approximation with two terms and that with only a single term, it is evident that the single term approximation is the more accurate one in the range we are interested in. The phase is therefore approximated here to be independent of *H*

$$\text{arg}\left[G^{*}\left(\omega_{0}\right)\right]\approx \frac{\pi }{2}\gamma$$ (23)

**Fig. 5.**
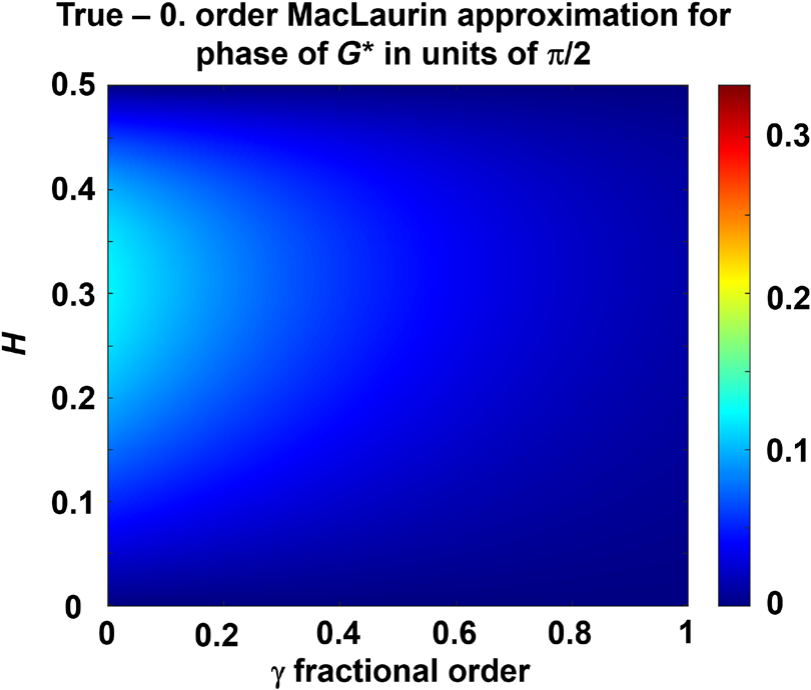
Absolute error in the approximation of the phase angle of the shear modulus. That is, the difference between Eqs. 22 and 20 normalized by π2.

The difference between this approximation and the exact result is plotted in Fig. 5. The error is small for most combinations of γ and *H*. This result is valid for a single frequency, ω_0_. Overall, the bandwidth used in each of our experiments is below one decade. We therefore make the additional assumption that the result of Eq. 23 is valid only within a narrow bandwidth around the center frequency ω_0_. The two steps of the inversion method for finding the fractal properties of the medium are:

1. Estimate the fractional order, γ, of the spring-pot describing the background material from the phase of the complex shear modulus using Eq. 23.

2. Estimate the effective Hurst index, *H*, from the power law exponent κ of the wavelength’s variation with frequency, Eq. 19, using the value for γ found in step 1.

### Phantom experiments (gel/fractal)

Shear wave vibrations were induced by using a custom-built setup where a cantilever assembly connected to a linear motor, source of the vibrations, is used to generate vibration inside the sample holder through a toothpick. The acquisition was performed using a multislice, single spin echo MRE sequence [Echo Time (TE) = 26 ms; Repetition Time (TR) = 1600 ms; field of view (FOV), 32 mm; matrix, 64 × 64; one average; six wave phases; 18 slices; and isotropic resolution, 0.250 mm)] with nine vibration frequencies 100, 150, 200, 250, 300, 350, 400, 450, and 500 Hz.

### Histology on human samples

Ethical approval was granted by the local ethics committee (CEERB PARIS NORD IRB00006477, protocol number CER-2022-168). A representative paraffin block of each tumor was selected by a pathologist. Full sections of 3 μm were cut on charged slides (Superfrost slides; Thermo Fisher Scientific, Waltham, MA, USA) and were immunostained with anti-CD31 antibody (JC/70A, 1:200, Dako, Glostrup, Denmark) to assess the vascularization of the tumor. Subsequently these sections were scanned at 20× magnification with an Aperio slide scanner.

The high-resolution histology images obtained (20,000 × 30,000 pixels, 0.4959 μm per pixel) were divided into smaller tiles of 1024 × 1024 pixels to examine different tissue sections individually and isolate irregularities to specific tiles (e.g., tears in tissue and areas of poor staining). A vessel extraction algorithm was applied to the tiles stained with CD31, converting them into binary images to perform box counting and calculate the Hurst index.

### Human experiments

Informed consent was obtained from all the healthy subjects. Imaging was performed on a 3-T PET-MRI full body system (Siemens Healthineers, Erlangen, Germany) using a multislice gradient echo sequence (Fast Field Echo TE = 9.4 ms; TR = 90 ms; eight slices; isotropic resolution of 4 mm^3^; and 25° flip angle) using a custom-built transducer for shear wave generation (*47*). Each individual scan consisted of four breath holds of approximately 15 s. Five different excitation frequencies were applied (40, 45, 50, 55, and 60 Hz). The MRE data were processed in Fourier space to remove high-frequency noise using a low-pass Blackmann Harris filter and were subsequently reconstructed as in Sinkus *et al.* (*48*). This allowed to calculate the complex shear modulus G^*^ = *Gd* + *iGl*, where *Gd* is the shear stiffness and *Gl* is the shear viscosity, and the phase angle $Y=\frac{2}{\pi }a\text{tan}\left(\frac{\mathrm{Gl}}{\mathrm{Gd}}\right)\in \left[0,1\right]$.

### Mouse experiments

All experimental procedures were performed in accordance with the European Community Council Directive and approved by the institutional committee: “Comité d’éthique en matière d’experimentation animale” under the protocols APAFIS#29203-2021011811346684 v5 and APAFIS#27258-2020090716004279 v6.

Imaging was performed on a 7-T MRI preclinical scanner (Bruker, Ettlingen, Germany; gradient strength, 660 mT/m) using an 8.6-cm body coil for transmission and a 2-cm surface coil for reception. The imaging was performed under anesthesia, induced with isoflurane delivered via a nose cone, constantly monitoring the respiration rate. Shear wave vibrations were induced by using a custom-built bed with the head fixated in a cantilever assembly connected to a linear motor, source of the vibrations. The acquisition was performed using a multislice, single spin echo MRE sequence (TE = 26 ms; TR = 1600 ms; FOV, 19.2 mm; matrix, 64 × 64; one average; six wave phases; 18 slices; and isotropic resolution, 0.250 mm) with four vibration frequencies 600, 700, 900, and 1000 Hz (*49*). Data were processed in a similar manner to the human experiments.

### Vessels immunohistochemistry on mouse brain

On the day of euthanasia, mice were transcardially perfused with heparinized saline, followed by 4% paraformaldehyde (PFA) in 0.1 M phosphate buffer, pH 7.4. Brains were removed, postfixed overnight in PFA, and cryoprotected in 20% sucrose. Coronal 30-μm-thick sections were cut frozen using a cryostat CM 1950 (Leica Biosystems, Nussloch, Germany).

Thirty-micrometer-thick floating coronal sections were incubated with primary antibody overnight at 4°C: Anti-glucose transporter-1 (Glut-1) (1:500, Millipore, Burlington, MA) was used to detect vessels. Fluorescent-labeled secondary Cy3 goat anti-rabbit immunoglobulin G (1:400, Jackson Immuno Research Laboratories) was applied for 1 hour at room temperature.
